# Substantial remission of prostate adenocarcinoma with dendritic cell therapy APCEDEN^®^ in combination with chemotherapy

**DOI:** 10.2144/fsoa-2019-0086

**Published:** 2019-10-29

**Authors:** Chaitanya Kumar, Jasmine Bagga, Srikanth Chiliveru, Sakshi Kohli, Asmi Bharadwaj, Minish Jain, Shriram Inamdar, Bandana Sharan

**Affiliations:** 1 R&D, APAC Biotech Pvt Ltd, Gurgaon, India; 2 Medical Oncology, Ruby Hall Clinic, Pune, India; 3 Xylem Clinical Research Pvt Ltd, Pune, India

**Keywords:** APCEDEN^®^, dendritic cells, immunotherapy, IFN-γ, neutrophil lymphocyte ratio, platelet lymphocyte ratio, prostate adenocarcinoma, regulatory T cells

## Abstract

Of the most prevalent solid tumors with advanced disease, prostate and ovarian cancer and non-small cell lung carcinoma have the fewest therapeutic options. Herein, we report the case of a 63-year-old male with metastatic prostate adenocarcinoma showing substantial remission post-administration of personalized dendritic cell-based vaccine APCEDEN^®^ in combination with chemotherapeutic drug Mitoxantrone. Therapeutic response displayed an interesting clinical correlation validated by PET scan images showing decreased fluorodeoxyglucose (FDG) avidity in the prostate gland, reduced skeletal metastases further established by the drop in serum Prostate Specific Antigen (PSA) levels and expression of immune assessment markers (IFN-γ, Tregs, neutrophil lymphocyte ratio and platelet lymphocyte ratio). This case demonstrates the potential efficacy of dendritic cell immunotherapy, showing a potent antitumor activity by enhancing the host immune responses, and improving quality of life.

## Lay abstract

Prostate adenocarcinoma is the most common cancer and second leading cause of cancer-related death in men. Advanced cancers have very few therapeutic options. Understanding of the immune system has led to the development of novel personalized vaccines as an emerging and efficient treatment modality for cancer. This case study describes the substantial remission of advanced prostate cancer after receiving the personalized dendritic cell therapy APCEDEN in combination with the chemotherapy drug mitoxantrone, even after the patient’s previous failed treatment history of standard hormonal, chemo- and radiotherapy regimens. This case stands as an interesting example of combination therapy for cancer benefiting the patient.

Globally, prostate, lung and colorectal cancers account for 42% of all cancer cases in men. Prostate cancer accounts for almost one in five new diagnoses [1]. Although in India the annual percentage change indicates an increasing trend in cancer incidence rates over time, over the past two decades the American Cancer Society’s annual statistics report a decline in the death rate resulting from cancer in the USA [2–4]. With an increase in available treatment options, survival for patients with metastatic castration-resistant prostate cancer (mCRPC) has improved significantly [5]. Five new drugs have been approved since 2010 and modern antiandrogen therapies along with immunotherapy have further helped to decrease the impact of this disease [6].

Active immunotherapy utilizing dendritic cells (DCs) loaded with tumor antigen *ex vivo* results in eliciting tumor-specific T-cell-mediated tumor cell toxicity. Antigen-loaded DCs have been tested in multiple clinical trials as therapeutic vaccines [7,8]. Varied formulations of DC vaccines utilizing alternative sources of tumor-associated antigens along with other adjuvants are also under experimentation to target early stages of tumor development.

The US FDA has approved use of Sipuleucel-T for the treatment of minimally symptomatic or asymptomatic metastatic hormone-refractory prostate cancer, which involves *ex vivo* loading of DC precursors with recombinant prostatic acid phosphatase fused to GM-CSF [9,10]. DCVAC/PCa is another DC-based vaccine under the Phase III clinical trial that utilizes the killed PSA-positive prostate cancer cell line (LNCaP). DCVAC/PCa has been shown to improve overall survival (OS) in patients, which might be due to enhanced PSA-specific T-cell responses along with downregulation of Treg cells [11]. Recently, rilimogene galvacirepvac (PROSTVAC; Bavarian Nordic A/S), which consists of a recombinant vaccinia vector prime followed by multiple boosts with a recombinant fowlpox vector plus transgenes for PSA and three costimulatory molecules, was shown to be efficacious to treat prostate cancer. A Phase II trial with 122 mCRPC patients with rilimogene galvacirepvac versus placebo group demonstrated an improvement in median OS of 8.5 months along with a 44% reduced death rate [12]; however, a recent study reported PROSTVAC demonstrated no effect on the OS of mCRPC patients in a Phase III trial [13]. Here, we present a case of metastatic prostate adenocarcinoma in which the patient received DC-based autologous immunotherapy, APCEDEN, and was successfully treated. A Phase II trial with the same DC vaccine APCEDEN highlighted its potency in 51 subjects with various cancer indications resulting in a response rate of 28.9 and 42.1% by RECIST and irRC respectively, and median time to progression as approximately 9 weeks [14].

## Patients & methods

### APCEDEN^®^ vaccine preparation

APCEDEN is an autologous DC formulation in which DCs are derived from CD14^+^ blood monocytes as previously described by Romani *et al.* [15] and loaded with whole-tumor lysate. In brief, the process begins with separation of peripheral blood mononuclear cells by apheresis and further isolation of monocytes from apheresis harvest by plastic adherence; culturing in Roswell Park Memorial Institute 1640 media (Lonza, NJ, USA) supplemented with cytokines IL-4 and granulocyte macrophage colony-stimulating factor (R&D Systems, MN, USA) and autologous plasma *in vitro*; and exposure of the patient’s own tumor tissue lysate on the sixth day. For antigen loading of DCs, ultrasound-guided 11 core CA prostate biopsy was collected in tissue transported medium (Nutriprep), and was further histopathologically confirmed for its malignancy. Tissue weighed 210 mg and tumor lysate was prepared by the freeze–thaw procedure as described by Nestle *et al.* [16], with protein concentration determined according to Bradford’s protein assay [17]. On the sixth day, 5 μg/ml of polyinosinic: polycytidylic acid (PolyI:C) (InvivoGen, CA, USA) was used as maturation stimuli; after 3 h of adding poly I:C, 1–20 μg/ml protein was loaded on DCs. Mature DCs were harvested on the eighth day and packaged as six doses (4–5 million mature DCs per dose) after stringent quality control by phenotypic, viability and sterility assessment. Phenotypic assessment involved checking for lineage and maturation markers for DCs (CD80/CD83/CD86/Anti-HLA-DR) as shown in Supplementary Figure 1 for the patient reported in this case report. Mature DCs were analyzed on FACS Calibur (BD Bioscience) after staining with CD83 (FITC), CD80 (PE), CD86 (APC), HLADR (PE) and 7AAD (PerCP) were compared with the unstained population and immature DCs harvested on sixth day were used as a control. Viability assessment was performed using 7AAD, and the viability of the cells was 90% with an expected 5–10% loss due to freeze and thaw procedures prior to infusion. Each dose of the vaccine is divided and administered via intravenous and intradermal routes. Six doses of APCEDEN were given at 15 day intervals (fortnightly) in a time frame of 3 months.

### Vaccine storage & logistics

APCEDEN vaccine is cryopreserved before infusion in the vapor phase of liquid nitrogen in a -196°C cryotank, and is transported in a portable liquid nitrogen tank conditioned to maintain the cryo temperature (-120 to -196°C).

### Neutrophil lymphocyte ratio & platelet lymphocyte ratio

The haemogram assessment of the patient was performed at pre-APCEDEN (baseline) and the remaining five doses of APCEDEN therapy to determine neutrophil lymphocyte ratio (NLR) and platelet lymphocyte ratio (PLR). The NLR was defined as a simple ratio between the absolute neutrophil count and the absolute lymphocyte count; similarly, PLR was defined as the ratio of the absolute platelet count and the absolute lymphocyte count [18,19].

### Treg & IFN-γ

The peripheral blood samples were drawn at baseline, dose 4 (midway through APCEDEN treatment) and dose 6 (end of APCEDEN therapy) of APCEDEN therapy and analyzed for Tregs and IFNγ. The flow cytometric gating strategy followed was CD3/CD4 and CD8/CD25/CD127 for T regulatory cells and CD3/CD4 and for IFN-γ, respectively [20,21].

## Case presentation

**Figure 1.**
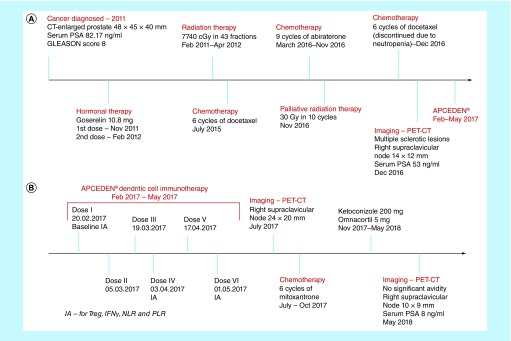
Treatment timeline. **(A)** The treatment timeline from the date of diagnosis until APCEDEN immunotherapy. **(B)** The APCEDEN treatment and postimmunotherapy regimen to show disease course. IA: Immune assessment; NLR: Neutrophil-to-lymphocyte ratio; PLR: Platelet-to-lymphocyte ratio; PSA: Prostate specific antigen.

A 63-year-old male was diagnosed with prostate adenocarcinoma. Upon diagnosis in 2011, PET scan revealed an enlarged prostate gland measuring 48 × 45 × 40 mm and a high serum PSA level of 82.17 ng/ml with a GLEASON score of 8, and classified as T1cN1M1b stage of prostate cancer. He was given neoadjuvant hormonal therapy in the form of Inj Goserelin 10.8 mg twice (first dose in November 2011 and second dose in February 2012), which resulted in complete resolution of bilateral iliac and para-aortic nodes. A second dose of hormone therapy was administered prior to radiation therapy. In view of organ-confined disease, he underwent radiation therapy for 14 months (7740 cGy in 43 fractions–4500 cGy in 25 fractions and 3240 cGy in 18 fractions). In July 2015, six cycles of chemotherapy in the form of docetaxel (taxotere) were administered as the first choice of the oncologist for the patient’s hormone-refractory metastatic prostate cancer treatment to reduce the observed post-radiation tumor progression to lymph nodes and skeletal metastasis. This was followed by abiraterone from March 2016 to November 2016, which is an effective therapy option primarily intended to treat metastatic prostate cancer that has metastasized to other parts of the body. In November 2016, he received ten cycles of palliative radiation therapy (30 Gy) to the pelvic region to alleviate obstructive urinary symptoms, hematuria, tenesmus, and pain. In December 2016, he was re-administered with docetaxel, in view of the earlier good response, which was stopped as he developed neutropenia and increased the risk of infection. Post-docetaxel treatment in December 2016, the PET-CT impressions reported multiple PSMA avid sclerotic lesions representing skeletal metastases and avidity in mediastinal, bilateral hilar and right supraclavicular nodes. Although there was no obvious PSMA avid lesion seen in prostate, the serum PSA level was still high at 53 ng/ml. The course of the treatment until immunotherapy is explained in Figure 1A. After consulting his oncologist, he chose to receive APCEDEN, an autologous dendritic cell immunotherapy, and the first and only approved immunotherapeutic cell-based product by CDSCO, the Central Drugs Standard Control Organization, which is the regulatory authority for Indian pharmaceuticals and medical devices. The APCEDEN treatment and post-immunotherapy regimen showing the disease course is presented in Figure 1B.

## Results

**Figure 2.**
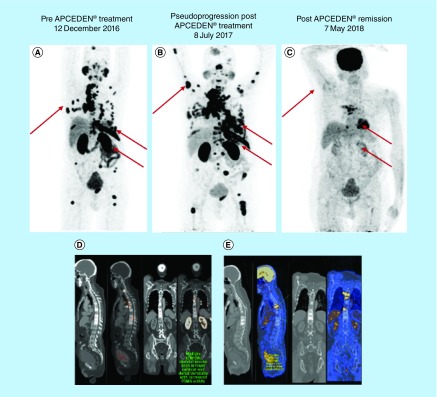
PET-CT scan of patient before APCEDEN treatment and after successfully completing six doses of the dendritic cell immunotherapy regimen. **(A–C)** Full body PET scan images comparing the patient’s metastasis, pseudoprogression (2 months after last dose of APCEDEN) and substantial remission of metastatic lesions (12 months after last dose of APCEDEN), respectively. Changes in tumor size is indicated by red arrows in the image. **(D & E)** PET scan image of vertebrae exhibiting decrease in metastatic lesions post-APCEDEN therapy.

**Figure 3.**
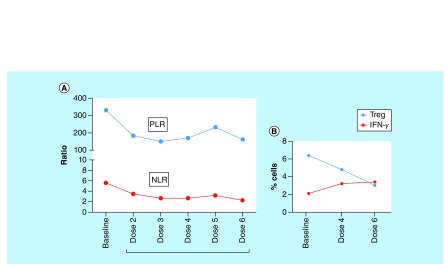
Immune assessment of the patient pre-APCEDEN (baseline) and during the course of treatment. **(A)** NLR and PLR values at baseline and all the six doses of immunotherapy regimen. **(B)** Immune assessment for IFN-γ and Treg positive cell populations was done by six color ATTUNE flow cytometer (ThermoFisher Scientific) at three time points: baseline, fourth dose and sixth dose of APCEDEN. Peripheral Blood was collected in EDTA vaccutainer (BD Bioscience) further processed and stained. The gating strategy for IFN-γ is CD3^+^/CD4^+^/IFN-γ and for Treg is CD3^+^/CD4^+^/CD25^+^/CD127 low. NLR: Neutrophil lymphocyte ratio; PLR: Platelet lymphocyte ratio.

Post the APCEDEN immunotherapy regimen of six doses administered fortnightly from February 2017 to May 2017, PET-CT scan in the following May 2018 did not reveal any appearance of significant avidity in the prostate gland with more than 60% reduction in size of the lesions in the left para-aortic, mediastinal and right supraclavicular nodes. Pseudoprogression of the disease was observed as a corollary of effective immunotherapy shortly after APCEDEN treatment, demonstrated by the increase in size and number of FDG avid metastatic lesions in the PET-CT image in July 2017 (Figure 2A–C). Remarkable remission of skeletal metastases as indicated by FDG avid sclerotic skeletal lesions was seen in D4, D5, D6 and D9 vertebral bodies as compared with the PET scan done before DC therapy in which significant avidity in vertebrae (C3, C6, D2, D4, D5, D6, D7, D8, D9, D10, D11 and D12 vertebrae) was observed (Figure 2D–E). Furthermore, no significant avidity in PET scan was observed in bilateral pelvic bones, bilateral femur, ribs, sternum, scapulae and skull bones when compared with the pre-immunotherapy scan. Positive response to immunotherapy was also supported by a lower serum PS level of 8 ng/ml post-APCEDEN. Reduction in NLR and PLR are associated with treatment efficacy [18,22]. The NLR and PLR for the patient were also seen to decrease during the course of the therapy (Figure 3A). To attribute the improvement in the patient to immunotherapy, the immune assessment of the patient was done at baseline and after dose 4 and 6, which showed an increased amount of IFN-γ-expressing CD4^+^ T lymphocytes along with a decrease in CD4^+^ and CD8^+^ Tregs in peripheral blood (Figure 3B). However, post-immunotherapy the patient received six cycles of mitoxantrone, an ‘antineoplastic’ or ‘cytotoxic’ chemotherapy drug, from July 2017 to October 2017, as he had an advanced hormone-refractory prostate cancer due to hormonal therapy failure earlier in the treatment history. Further, he was administered ommnacortil 5 mg (corticosteroid), ketoconizole 200 mg from November 2017 until May 2018. This case study is an interesting example of combination therapy benefiting the patient.

## Discussion

Immunotherapy along with modern antiandrogen therapies has the potential to severely impact the survival of patients with prostate adenocarcinoma. With the success of the first cancer vaccine, there are increased efforts to develop novel prostate cancer vaccine candidates including DNA subunit vaccines, *ex vivo* antigen-loaded DCs and viral-based vector systems targeting specific tumor-associated antigens. DCs are identified as key players of immunotherapy as they present antigens to T cells and link the innate and adaptive immune system. Antigen-loaded DC-based therapeutic cancer vaccines have been tested and proven successful in multiple clinical trials in patients with several different types of cancers, including melanoma, malignant lymphoma and prostate cancer, thereby suggesting increased antitumor immunity. For the success of DC vaccines, it is very important to take notice of selecting the personalized tumor-specific antigen by using patient-specific biopsy homogenates, efficient loading of antigen onto DCs for presentation to the activated T cells and preparation of the correct number of DCs in each dose, along with route of infusion of DCs into the patient.

The autologous DC vaccine APCEDEN has been reported as safe and shown significant improvement in the quality of life of cancer patients. A clinical trial conducted in India described OS benefit of 397 days for patients with solid malignancies after receiving APCEDEN treatment [14,23,24]. Here, we describe a case of prostate cancer with metastases. The patient showed positive response to hormone therapy (goserelin) but developed metastatic lesions, which were significantly reduced in size after receiving immunotherapy. Goserelin is a LHRH analog considered to be effective for the treatment of prostate cancer by successfully maintaining persistent suppression of serum testosterone resulting in castration with a median follow-up of 41 weeks [25]. Hormone therapy followed by immunotherapy in combination with mitoxantrone chemotherapy resulted in significant reduction in disease progression, thus emphasizing the need for combinatorial approaches for cancer treatment. Immune assessment before beginning the immunotherapy and during the treatment course revealed positive changes such as decrease in NLR and PLR, indicating improved cell-meditated immunity as the lymphocytes percentage increased [18]. Correspondingly, increase in number of IFN-γ-expressing CD4^+^ T cells played a critical role in expansion and regulation of CD8^+^ T cells, consequently augmenting the antitumor effects of cell-mediated immunity [26–29]. Furthermore, the number of Tregs was diminished thereby reducing their immune suppressive effect [30–33], suggesting APCEDEN as a promising immunotherapeutic modality for cancer. The increased immune responses can sometimes lead to ‘pseudoprogression’, which is the radiologic appearance of an increase in tumor burden with subsequent tumor regression or response. Biologically, pseudoprogression is not actual tumor growth, but rather results from infiltration of inflammatory cells, edema and necrosis generated by immunotherapy [34–37]. Pseudoprogression was observed in this case study soon after the completion of DC therapy, which was further decreased as evident in the latest PET scan images. Hormone therapy using LHRH analogs have been reported to exhibit side effects such as gastrointestinal symptoms (nausea, vomiting and abdominal pain) and decreased bone density, which result in a decreased quality of life after treatment. In such cases, immunotherapy such as APCEDEN are of prime importance as they have been shown to improve the survival and quality of life score of the patients [38]. Similar to the recently reported Gliovax Phase II trial on integration of DC therapy into a standard radiochemotherapy regimen for newly diagnosed glioblastoma [39], this case study illustrates the benefits of immunotherapy in prostate cancer patients when used as an adjunct therapy to existing line of treatment regimens after a brief pseudo progression.

## Future perspective

The current case report suggests autologous DC immunotherapy could form a safe, feasible and efficacious therapy in combination with the systemic chemotherapy drug mitoxantrone, providing a step forward toward an evolving chemoimmunotherapy approach for cancer treatment. DC vaccine optimization through effective antigen presentation strategies and operational combination therapy strategies are the foci of ongoing and upcoming studies.

**Summary points**

- APCEDEN^®^ is a personalized dendritic cells (DC)-based immunotherapy product that enhances antitumor immunity by *ex vivo* maturation of monocyte-derived DCs pulsed with whole tumor lysate.
- A 63-year-old Asian male diagnosed with prostate adenocarcinoma shows substantial remission post APCEDEN immunotherapy in combination with anthracenedione antineoplastic drug mitoxantrone.
- Therapeutic response shows reduction in disease progression validated by decrease in fluorodeoxyglucose avidity in prostate gland, remission of skeletal metastases and reduced serum prostate specific antigen level.
- This case study demonstrates the efficacy of APCEDEN Immunotherapy in combination with chemotherapy regimen resulting in a significant disease remission benefiting the patient.
