# Supplementary material for: Substantial remission of prostate adenocarcinoma with dendritic cell therapy APCEDEN® in combination with chemotherapy
Source: Future Sci OA. 2019 Oct 29;5(10):FSO435. doi: 10.2144/fsoa-2019-0086 (PMC6900976; doi:10.2144/fsoa-2019-0086)
Supplement: Supplementary file 1 [file fsoa-05-435-s1.pdf]

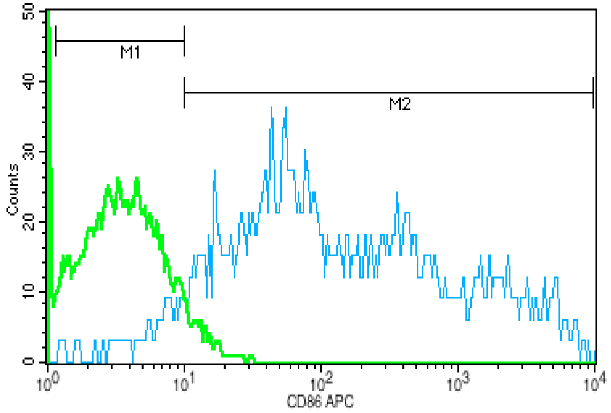

Histogram Statistics

Marker	Events	% Gated	% Total
All	11067	100.00	36.89
M1	741	6.70	2.47
M2	10314	93.20	34.38

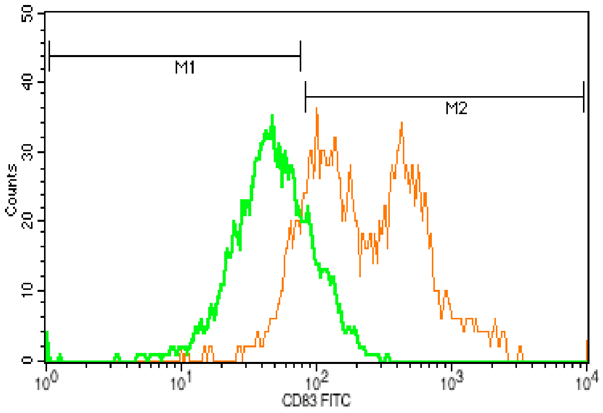

Histogram Statistics

Marker	Events	% Gated	% Total
All	7378	100.00	36.89
M1	978	13.26	4.89
M2	6212	84.20	31.06

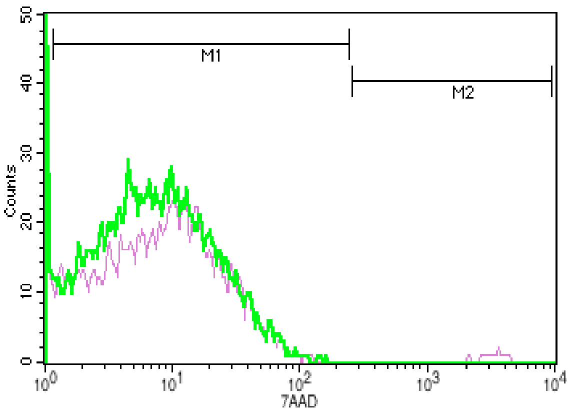

Histogram Statistics

Marker	Events	% Gated	% Total
All	7490	100.00	74.90
M1	6115	81.64	61.15
M2	72	0.96	0.72

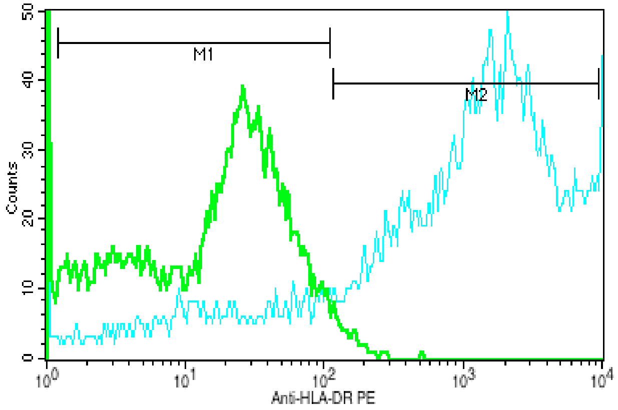

Marker	Events	% Gated	% Total
All	16000	100.00	100.00
M1	2725	17.03	17.03
M2	12207	76.29	76.29

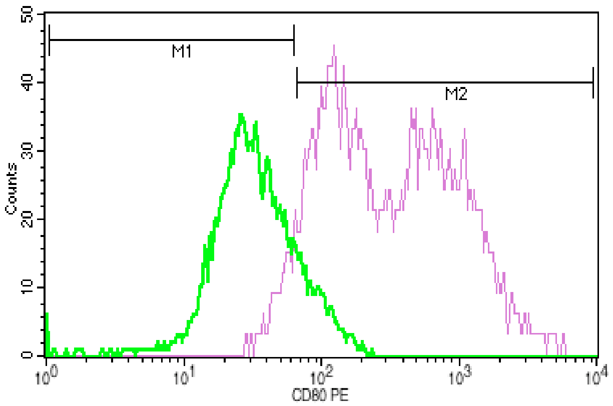

Histogram Statistics

Marker	Events	% Gated	% Total
All	11067	100.00	36.89
M1	687	6.21	2.29
M2	10281	92.90	34.27
